# Prediction of anemia using facial images and deep learning technology in the emergency department

**DOI:** 10.3389/fpubh.2022.964385

**Published:** 2022-11-09

**Authors:** Aixian Zhang, Jingjiao Lou, Zijie Pan, Jiaqi Luo, Xiaomeng Zhang, Han Zhang, Jianpeng Li, Lili Wang, Xiang Cui, Bing Ji, Li Chen

**Affiliations:** ^1^Medical School of the Chinese PLA, Beijing, China; ^2^Department of General Medicine, The First Center of the Chinese PLA General Hospital, Beijing, China; ^3^School of Control Science and Engineering, Shandong University, Jinan, Shandong, China; ^4^Luoyang Outpatient Department of 63650 Army Hospital of the Chinese PLA, Luoyang, China; ^5^Department of Orthopedics, Chinese PLA General Hospital, National Clinical Research Center for Orthopedics, Sports Medicine and Rehabilitation, Beijing, China

**Keywords:** anemia, deep learning, emergency medicine, facial recognition, diagnosis

## Abstract

**Background:**

According to the WHO, anemia is a highly prevalent disease, especially for patients in the emergency department. The pathophysiological mechanism by which anemia can affect facial characteristics, such as membrane pallor, has been proven to detect anemia with the help of deep learning technology. The quick prediction method for the patient in the emergency department is important to screen the anemic state and judge the necessity of blood transfusion treatment.

**Method:**

We trained a deep learning system to predict anemia using videos of 316 patients. All the videos were taken with the same portable pad in the ambient environment of the emergency department. The video extraction and face recognition methods were used to highlight the facial area for analysis. Accuracy and area under the curve were used to assess the performance of the machine learning system at the image level and the patient level.

**Results:**

Three tasks were applied for performance evaluation. The objective of Task 1 was to predict patients' anemic states [hemoglobin (Hb) <13 g/dl in men and Hb <12 g/dl in women]. The accuracy of the image level was 82.37%, the area under the curve (AUC) of the image level was 0.84, the accuracy of the patient level was 84.02%, the sensitivity of the patient level was 92.59%, and the specificity of the patient level was 69.23%. The objective of Task 2 was to predict mild anemia (Hb <9 g/dl). The accuracy of the image level was 68.37%, the AUC of the image level was 0.69, the accuracy of the patient level was 70.58%, the sensitivity was 73.52%, and the specificity was 67.64%. The aim of task 3 was to predict severe anemia (Hb <7 g/dl). The accuracy of the image level was 74.01%, the AUC of the image level was 0.82, the accuracy of the patient level was 68.42%, the sensitivity was 61.53%, and the specificity was 83.33%.

**Conclusion:**

The machine learning system could quickly and accurately predict the anemia of patients in the emergency department and aid in the treatment decision for urgent blood transfusion. It offers great clinical value and practical significance in expediting diagnosis, improving medical resource allocation, and providing appropriate treatment in the future.

## Introduction

Anemia is characterized by a hemoglobin concentration below a specified cut-off point; this cut-off point depends on the age, gender, physiological status, smoking habits, and altitude at which the population being assessed lives. Current hemoglobin cut-off recommendations range from 13 to 14.2 g/dl in men and 11.6 to 12.3 g/dl in women (1). Severe anemia is often a sequela of malnutrition, parasitic infections, or underlying disease (2) and is also caused by trauma or other medical conditions such as gastrointestinal hemorrhage. In emergency departments, acute blood loss diseases often cause severe anemia, such as trauma, gastrointestinal hemorrhage, etc., and require quick identification and prompt restoration of the circulation volume to save the patients. Without immediate attention, patients will bleed to death from hemorrhagic shock (3). The classic symptoms of anemia are fatigue and shortness of breath, paleness of the mucous membranes and resting tachycardia (4). Interestingly, several reports have shown that anemia can be qualitatively associated with subjective assessment of the pallor in various parts of the body, such as the conjunctiva, face, lips, fingernails, and palmer creases (5–11). Previous studies have demonstrated that hemoglobin absorbs green light and reflects red light (12); hemoglobin concentration can affect tissue color.

Nowadays, a complete blood count is a common way to diagnose anemia. However, blood samples are obtained via invasive venipuncture, which necessitates the presence of professional medical staff and equipment (13–15). In the emergency department or ICU, obtaining information about blood hemoglobin levels is essential to ensure whether the patient needs an instant blood transfusion to save their lives (16). Thus, CBC may not be adequate or fast enough to meet the demand of doctors when screening for anemia patients fast and accurately, especially in mass casualty incidents such as war settings. With the rapid development of technology, noninvasive facial recognition technology has been widely used in medicine, such as the area of diagnosis of genetic disorder diseases diagnosis (17, 18), the area of diagnosis of dermatological diseases diagnosis (19, 20), the area of nervous system diseases (21, 22), etc. Researchers have been studying mucous membrane color changes as a potential biomarker for rapid and reliable anemia diagnosis using facial recognition technology in recent years (23–25).

Deep learning (DL), a subfield of artificial intelligence (AI), passes input through a large number of layers of interconnected nonlinear processing units to represent complicated and abstract concepts (26). Deep learning has had numerous important breakthroughs in fields as diverse as speech recognition, image recognition, natural language processing, translation, etc. (27–30). In this study, we will use the deep learning method to extract features from facial images and establish a correlation with the anemic state through layers of training.

Our study aims to determine whether the facial images taken under specific circumstances correlate with the anemic state. Researchers like Dr. Suner and Dr. Collings have found a model to detect the hemoglobin concentration from the analysis of conjunctiva (23, 24). Our goal is to develop a model to predict patients' anemic states using images of the patients with the analysis of the entire face taken from the portable pad so that it can promote fast and accurate screening for the anemic state of emergent and severe patients.

## Materials and methods

### Video collection

This was an observational prospective sample study. From October 1, 2021, to 13th April 2022, all videos were collected from patients in the critical care area in the emergency department of Chinses PLA General Hospital First Central Division for any chief complaint. The inclusion criteria were as follows: (1) 18 years old. The exclusion criteria were as follows: (1) patients or guardians unwilling to provide written consent, (2) Patients suffering from diseases affecting the color of the face except for blood loss (such as jaundice, skin diseases that affect the skin color, etc.), (3) known hypoxia (SpO_2_ <90%), and (4) receiving or due to receive a blood transfusion before video collection and blood sample measurement. All patients who participated provided their informed written consent.

All patients were asked to lie supine as comfortably as possible. Videos were made under the ambient indoor stable light with the camera of a pad (AIM75-WIFI). The pad was positioned 40 cm directly in front of the patients' faces to ensure that the whole face was captured on the screen. We shot a 5-s video with the pad placed in front of the faces; then, we rotated the pad 45 degrees to the left and right of the faces, with the distance unchanged, and shot another two 5-s videos. This way, we made a 15-s video of each patient who agreed to participate. The automatic focus was used throughout, and the flash was forbidden. Videos were captured in High Frame Rate 60 format and stored in Mp4 format. The resolution of the videos was 1,280 × 720. The blood sample of each participant was acquired immediately after the video, and hemoglobin measurement was carried out within 30 min of sample acquisition, ensuring that hemoglobin results matched the video analysis. Demographic information for patients included gender, age, admitting diagnosis, and hospital laboratory-reported hemoglobin results (Table 1). All videos and demographic information were collected by a single operator to reduce the variability. Research approval was granted by the Institutional Review Board (IRB) of the Medical Ethics Committee of Chinese PLA General Hospital.

**Table 1:** Demographic information of the patients in the research.

**Demographic information**	**Anemia patients** ***n* = 217**	**Non-anemia patients** ***n* = 99**	***P*-value**
Age (mean ± SD)	66.11 ± 17.51	59.00 ± 18.06	< 0.05
Male (%)	64.97%	57%	>0.05
Hemoglobin concentration (mean ± SD)	88.69 ± 20.64	142.39 ± 14.79	< 0.05
**Diagnosis distribution (%)**			
Motor system diseases	3.61%	2.02%	
Digestive diseases	41.01%	20.20%	
Cardiovascular system diseases	11.98%	33.33%	
Neural system diseases	7.83%	18.18%	
Genitourinary diseases	16.12%	3.03%	
Respiratory system disease	8.29%	11.11%	
Endocrine system diseases	1.38%	4.04%	
Multiple system diseases	9.67%	8.08%	

SD, standard deviation.

P < 0.05 with the statistical difference between the two groups.

### Procedure

**Figure 1:**
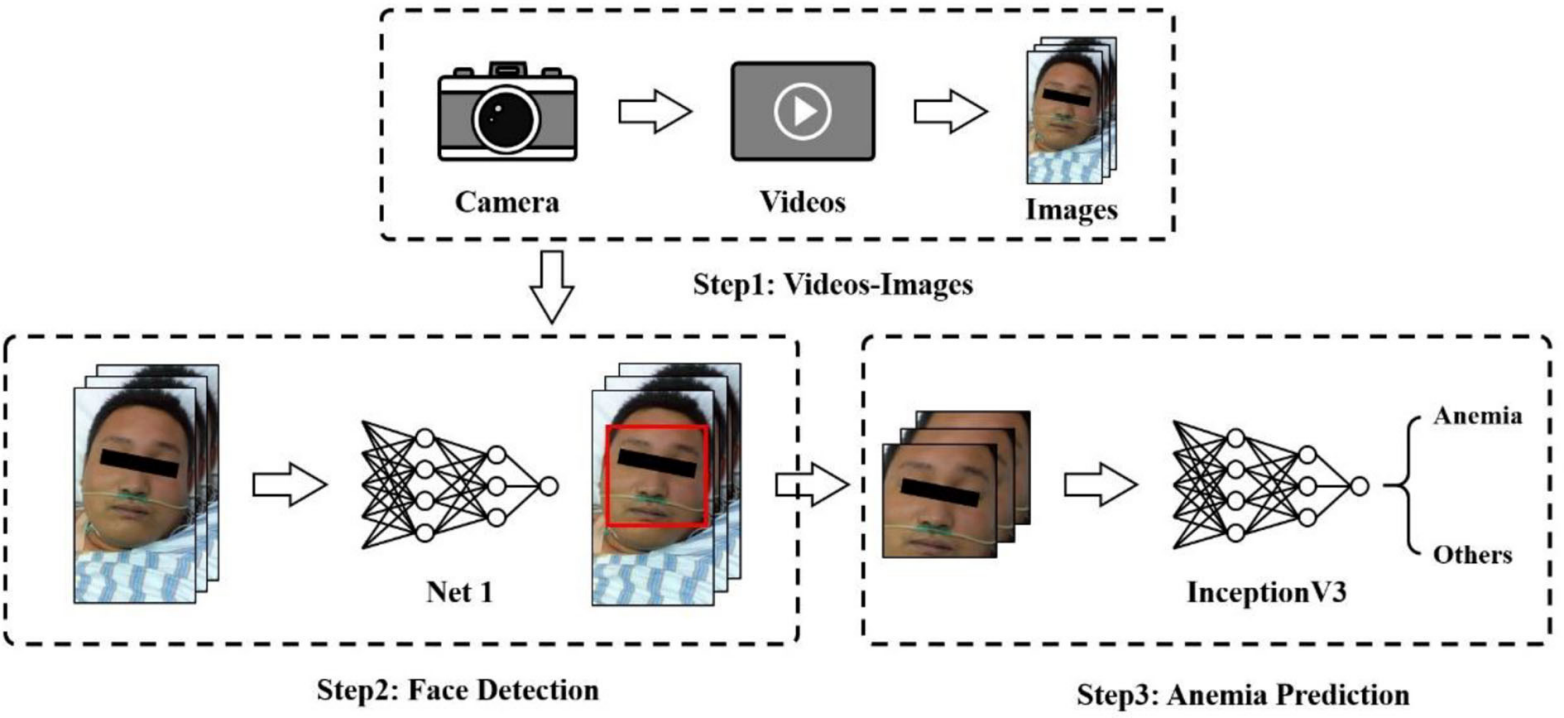
Procedure of anemia prediction.

This section presents our proposed framework for anemia prediction, as shown in Figure 1. The framework consists of three major modules: the video-image module, face detection network, and anemia prediction network. Patient videos were first fed into a video-image module and converted to images. Then, a face recognition algorithm was performed to produce the detected faces of patients, which were the inputs of the anemia prediction network.

### Data pre-processing

As anemia prediction relied on an image classification strategy, video frames were retrieved and saved as images. During shooting, patients could assume different positions. Face correction was performed to correct the face direction. Considering that there were hundreds of frames in a video, we chose to utilize some of them. Images were extracted and stored every 50 frames from videos of all patients, which finally built the dataset used in our experiments. Data augmentation, such as horizontal flipping, zooming, and rotation, was used to reduce overfitting and improve model performance.

In the study, we labeled the data at the patient level. All the patients in the research received a complete blood count test shortly after the video collection to determine whether they were anemic or not. Although one patient might have multiple videos, images extracted from videos have the same label if they belong to the same person. Specifically, if a patient was diagnosed with anemia, all images extracted from the videos of the patient were labeled as anemic or otherwise.

### Face detection

We used the service offered by Megvii Co., Ltd., known as Face++, as the face recognition and detection solution. Megvii is a Chinese technology company that mainly focuses on developing image recognition and deep learning software. Megvii manages one of the world's largest research institutes specializing in computer vision, and it is the largest provider of third-party authentication software in the world. Its product, Face++, is the world's largest open-source computer vision platform. The service released on their artificial intelligence open platform can detect and analyze human faces with the provided images. Even patients with different postures or expressions can produce results, which saves us quite a lot of time in annotating and training a face detector from scratch.

We used the Face++ detector to detect faces within images and got back face bounding boxes for each detected face. The values of the bounding boxes were used to crop and extract face regions from the original images. The images, after cropping, were down-sampled and resized into 224 × 224-pixels for subsequent anemia prediction.

### Anemia prediction

While most traditional machine learning methods depend largely on hand-crafted features, deep learning techniques have received sufficient attention for various computer science tasks, including classification, object detection, and segmentation. In this study, we compared and showed the classification results of five convolutional neural networks, including ResNet50, MobileNet, InceptionV3, EfficientNetB0, and DenseNet121 (Tables 2, 3) before finally choosing InceptionV3 as the proposed model.

**Table 2:** Five models' prediction results of three tasks at the image level.

**Results**	**Task-1**		**Task-2**		**Task-3**	
	**Accuracy**	**AUC**	**Accuracy**	**AUC**	**Accuracy**	**AUC**
MobileNet	79.70%	85.61%	69.10%	72.66%	70.20%	77.60%
ResNet50	78.26%	82.54%	73.10%	79.55%	67.70%	72.95%
DenseNet121	78.47%	83.17%	72.29%	77.52%	73.30%	81.72%
EfficientNetB0	69.98%	75.70%	69.70%	72.66%	72.85%	78.30%
InceptionV3	82.37%	84.02%	68.37%	69.79%	74.00%	82.10%

AUC, area under the curve.

**Table 3:** Five models' prediction results of three tasks at the patient level.

**Results**	**Task-1**			**Task-2**			**Task-3**		
	**Accuracy**	**Sensitivity**	**Specificity**	**Accuracy**	**Sensitivity**	**Specificity**	**Accuracy**	**Sensitivity**	**Specificity**
MobileNet	80.19%	90.74%	69.23%	71.33%	79.42%	63.24%	78.95%	88.47%	58.34%
ResNet50	82.08%	85.19%	78.85%	78.68%	90.21%	67.65%	78.95%	76.93%	83.34%
DenseNet121	81.14%	92.58%	69.23%	73.53%	85.30%	61.77%	73.68%	73.08%	75.00%
EfficientNetB0	78.31%	87.04%	69.23%	74.27%	77.94%	70.59%	81.58%	80.77%	83.33%
InceptionV3	84.02%	92.59%	69.23%	70.58%	73.52%	67.64%	68.42%	61.53%	83.33%

In addition to data augmentation, applying pre-trained models learned from large-scale datasets, such as ImageNet, was another way to reduce overfitting (31). In this study, we initialized the models for anemia prediction with pre-trained ImageNet weights and fine-tuned them on our own training dataset. Specifically, taking a 224 × 224-pixel image as input, the anemia prediction network was initially initialized with pre-trained ImageNet weights. We froze all layers and trained only the top layers until convergence. For this step, a relatively large learning rate (1e−3) was used. Then, we unfroze all the layers, fitted the model using smaller learning (1e−4), and saved the best model.

### Experimental settings

We conducted five-fold cross-validation with different seeds to evaluate our prediction method and reported the mean accuracy and AUC among five runs of combined test folds. For each experiment, we split the dataset into training and validation sets with a ratio of 7:3 at a patient level, which meant that images extracted from one person did not appear in different datasets, i.e., training or validation set. We set different class weights to solve the class imbalance problem that emerged in our research. Only the best model was saved based on the quantity monitor's valid accuracy. The model was validated on a holdout set, and the performance was assessed by sensitivity, specificity, accuracy, and area under the receiver operating characteristic curve (AUC). All codes were implemented in Tensorflow and run on an NVIDIA GeForce RTX 2080 Ti GPU.

### Clinician assessment

Two senior emergency department doctors were invited to subjectively assess the validation videos. Doctors were first shown three example videos of anemic patients and three videos of non-anemic patients. Then, all the videos in the validation set were shown randomly to the doctors. Doctors were asked to rank each video as “anemic” or “not anemic.” Each doctor's accuracy, sensitivity, and specificity were assessed to show the clinical performance in detecting anemia patients.

## Results

Our research recruited 362 patients, and 362 videos of faces were taken. Two experienced physicians were invited to assess the quality of the videos, and 45 videos were removed for a low definition or failure to display a complete face image. Thus, 316 face videos of 316 patients were used in the research, of which 217 patients were diagnosed with anemia based on the results of a complete blood count, and the average hemoglobin concentration was 10.55 g/dl. One hundred ninety-eight male and 118 female patients were included in the research, and the average age was 63.88. Demographic information of the patients is shown in Table 1. Three tasks were performed in the research. Task 1 aimed to predict the anemia of patients (Hb <13 g/dl in men and Hb <12 g/dl in women), while Task 2 aimed to predict the mild anemia of patients (Hb <9 g/dl). Finally, Task 3 aimed to predict the severe anemia of patients (Hb <7 g/dl).

**Figure 2:**
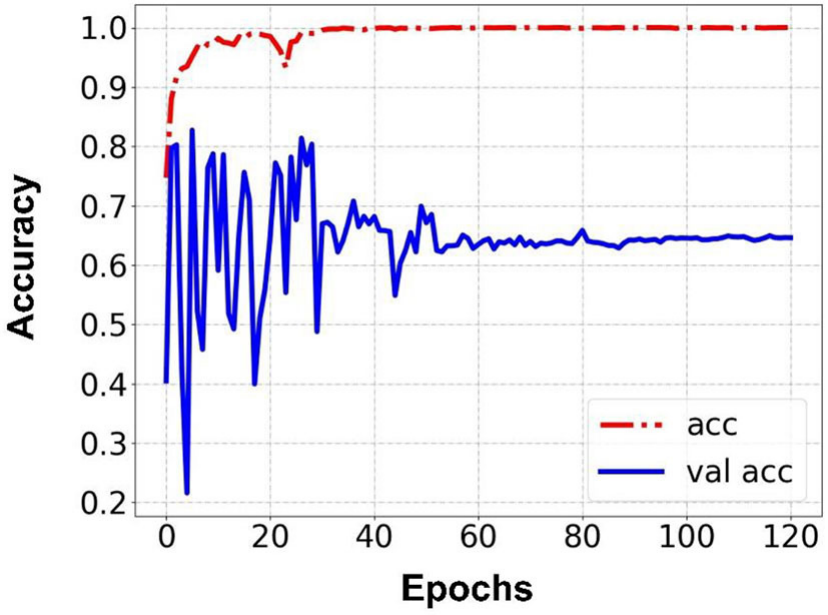
Accuracy for Task 1 in an image.

**Figure 3:**
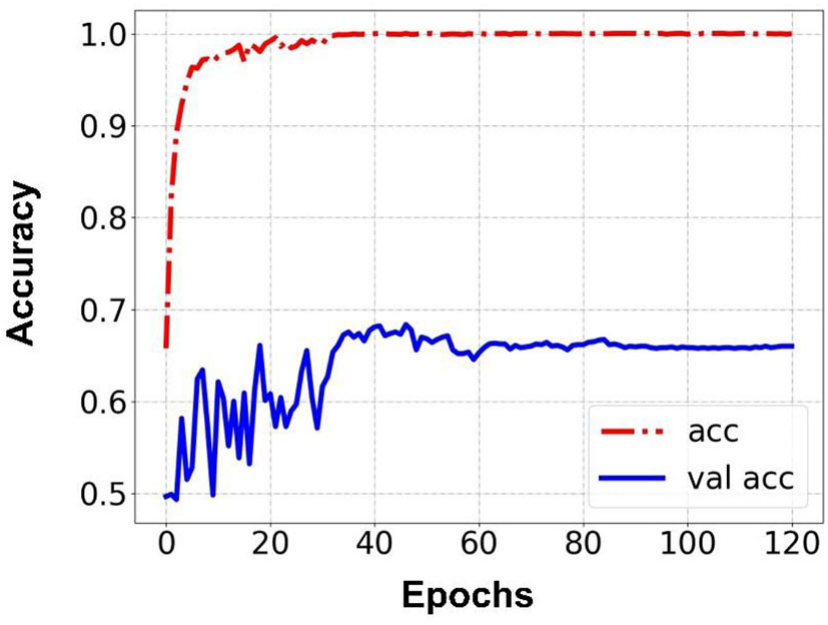
Accuracy for Task 2 in an image.

**Figure 4:**
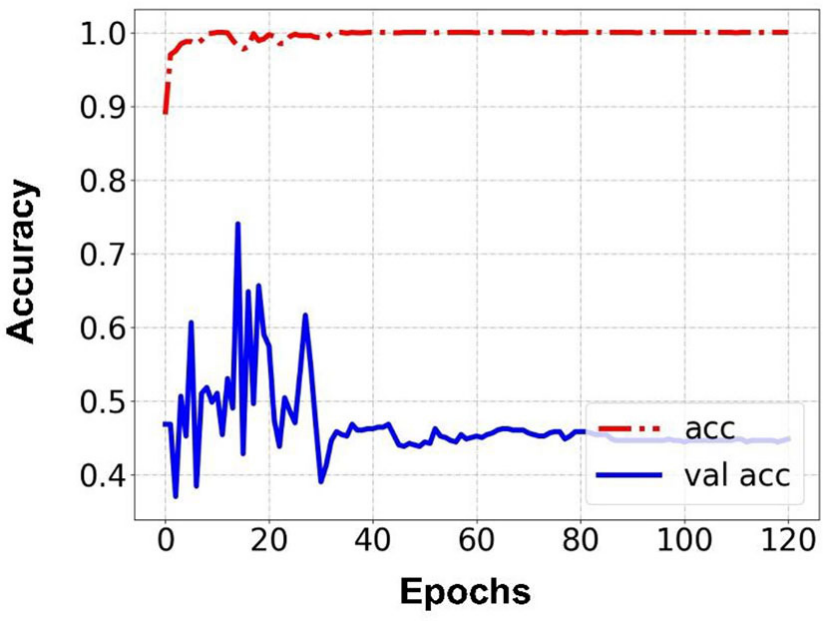
Accuracy for Task 3 in an image.

**Figure 5:**
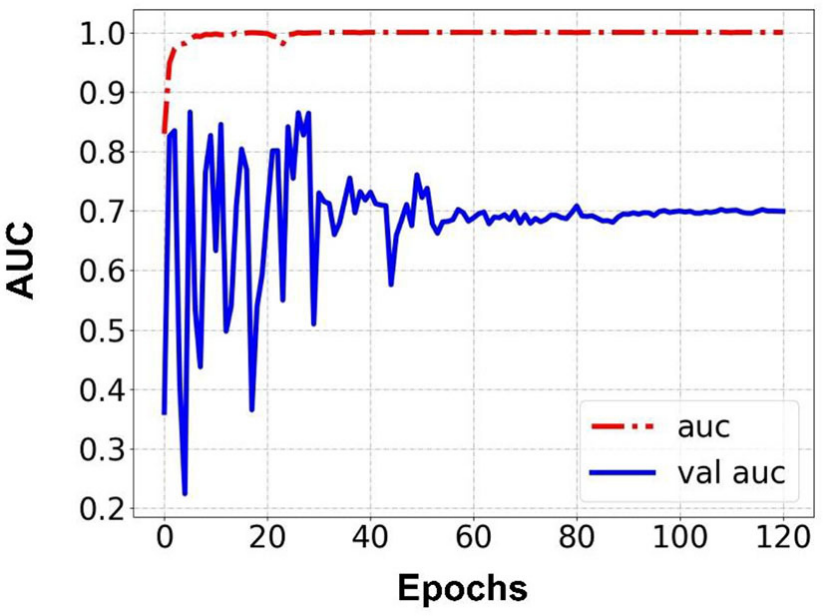
AUC for Task 1 in an image.

**Figure 6:**
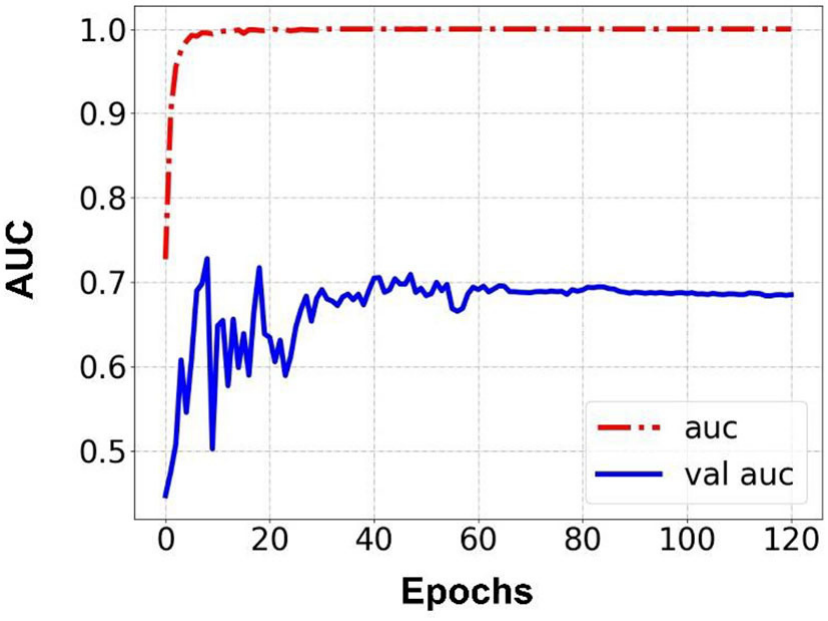
AUC for Task 2 in an image.

**Figure 7:**
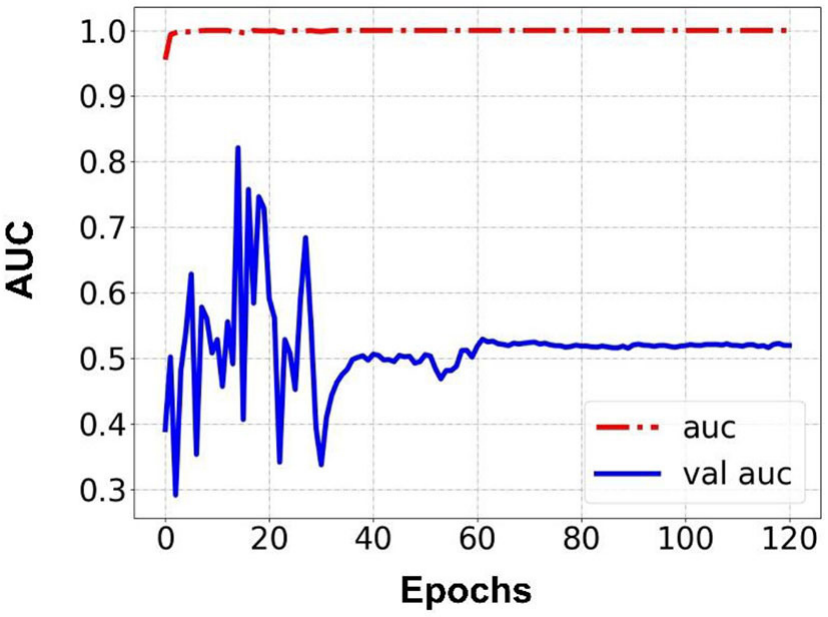
AUC for Task 3 in an image.

Prediction results were performed at two levels for each task: image and patient levels. With the help of data augmentation technology, 6,993 images of patients were extracted from the 316 videos and split into training data sets and validation data sets with a ratio of 7:3. The image level and patient level were two evaluation forms for the results. The image level and the prediction results of the model were assessed by the accuracy and AUC. Table 2 shows the results of three tasks at the image level. Figures 2–4 demonstrated the accuracy results for Task 1, Task 2, and Task 3 in image level, and Figures 5–7 demonstrate the AUC results for Task 1, Task 2, and Task 3. The patient-level results were the aggregating results of the image level and directly reflected the model's prediction ability in the clinical environment. Accuracy, sensitivity, and specificity were used to evaluate the prediction ability at the patient level, which is shown in Table 3.

To compare the performance of the prediction model and assess the clinical acceptability, we invited two senior doctors from the emergency department to assess each video in the validation set as anemic or not. The performance of each doctor was assessed by the accuracy, sensitivity, and specificity, and the comparison between the prediction model and the doctors' prediction is shown in Table 4.

**Table 4:** The comparison of the prediction model with clinical assessment.

	**Prediction model**	**Doctor 1**	**Doctor 2**
Accuracy	82.37%	55.23%	51.46%
Sensitivity	92.59%	66.61%	63.20%
Specificity	69.23%	37.50%	32.51%

## Discussion

Our research used the facial images of patients combined with the technology of deep learning to build the prediction model for the detection of anemia. The research performed three tasks to examine the model's ability to detect patients with varying anemia degrees. Two comparisons were made to evaluate the prediction model: the first comparison was between our model and Collings's model (23) and Hermoza's model (32). Collings's prediction was based on the conjunctiva and showed 76% accuracy in predicting anemia. Hermoza's model was based on the fingernail and showed 68% accuracy in predicting anemia. Meanwhile, our prediction model reached 84.02% accuracy, indicating that anemia showed better prediction ability than other prediction models. The second comparison was between our model and senior doctors. Both these doctors have less accuracy than the prediction model, scoring 55.23 and 51.46% accuracy, respectively, indicating the promising clinical utility of our model. All the results showed that the model could accurately predict the anemia patients with the facial images and had relatively good performance for predicting mild and severe anemia patients. In particular, for the severe anemia prediction, our results showed the promising performance of the model in the area of clinical treatment aid. The strengths and limitations of each will be discussed in the following paragraphs.

**Figure 8:**
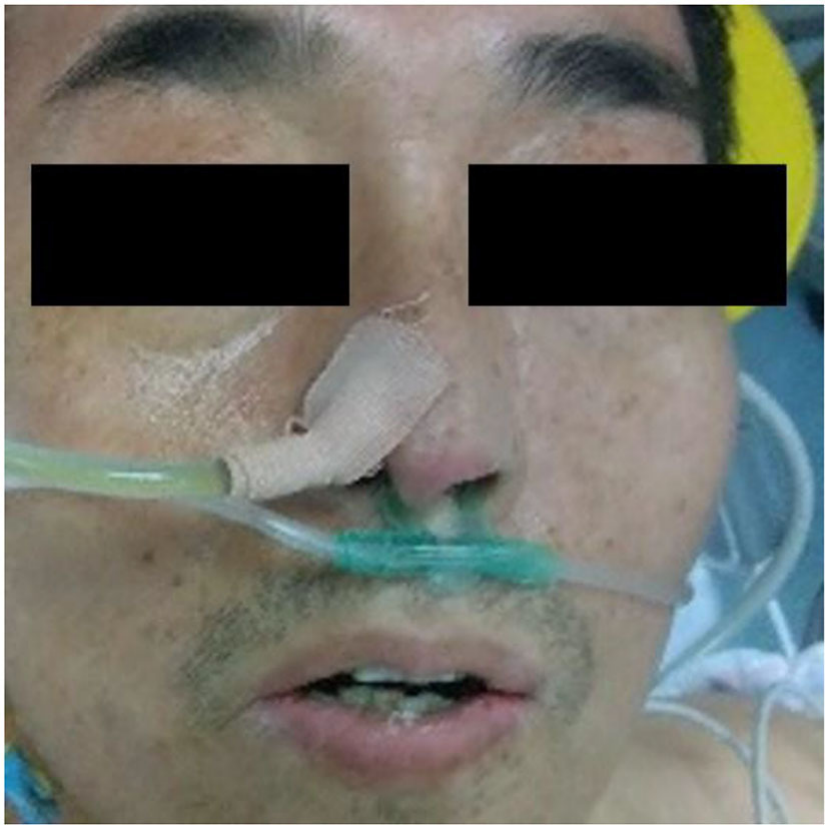
Original image of a patient with anemia.

**Figure 9:**
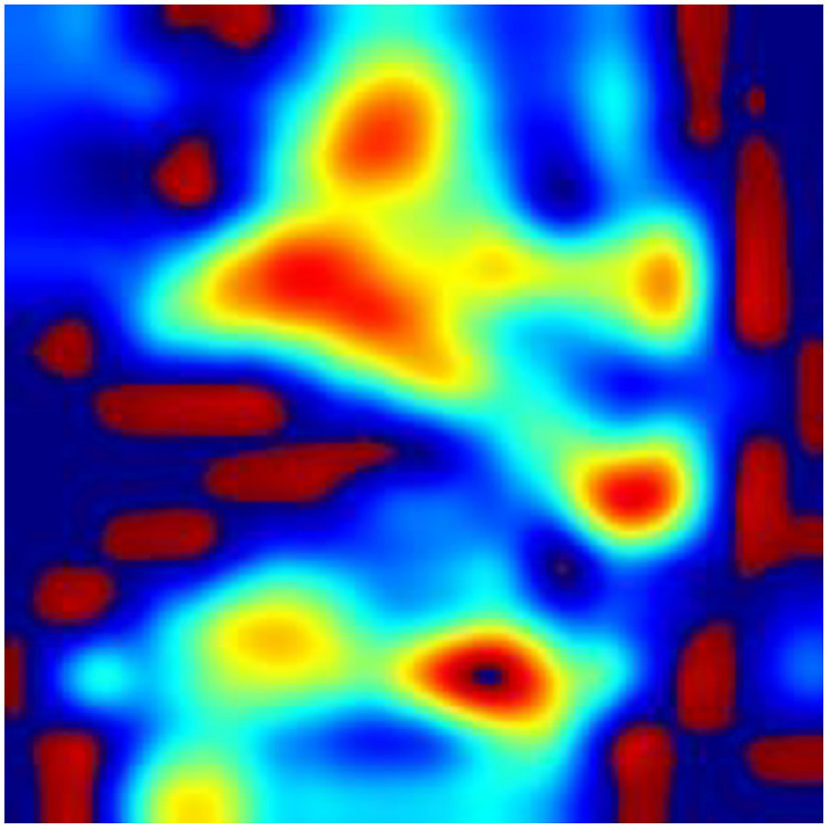
Support for the anemia category according to visualization of the Grad-CAM.

**Figure 10:**
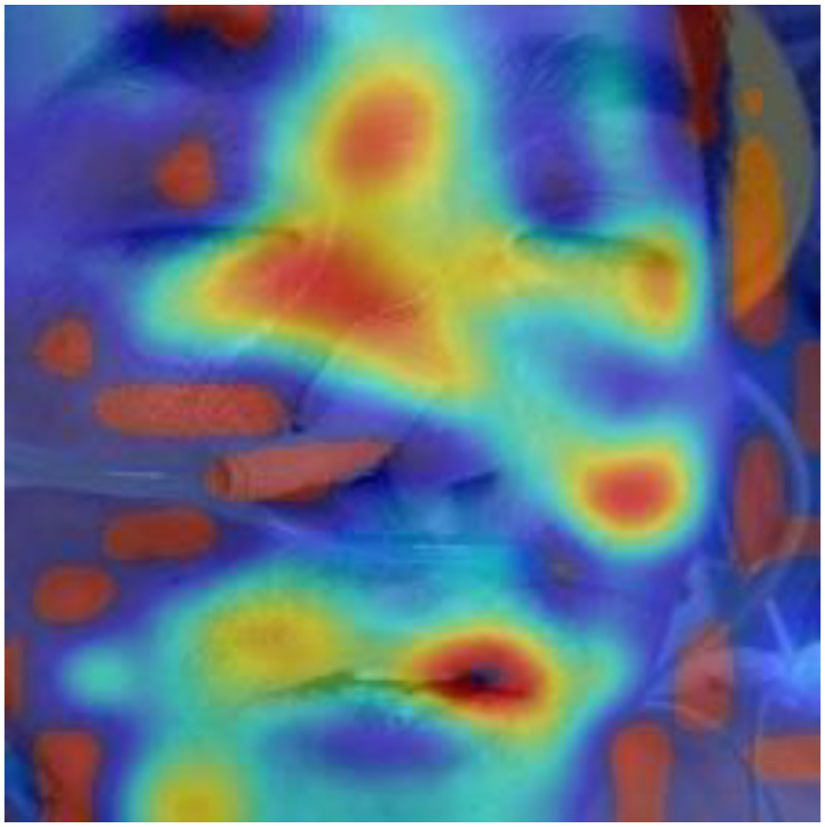
Fusion image of original image and Grad-CAM.

According to the WHO, anemia is a highly prevalent disease, affecting over two billion people worldwide (33). In recent years, rapid screening and diagnosis of anemic patients has become a hot topic. The theoretical support for the research is obvious: anemia may correlate with the pallor in various regions of the patient's body, such as fingernail beds, conjunctiva, palmar creases, and so on (7, 9, 10). The rapid development of new devices (34) combined with the theory leads to great achievement in the area of the noninvasive, quick detection of anemia. Learning from the reports about the detection of anemia in recent years, fingernail beds and conjunctiva are two important regions for detecting anemia. Fingernail beds and conjunctiva are short of melanocytes, which can affect the red light reflection of hemoglobin. Thus, they are of great use in anemia detection. However, it is worth noting that those researchers require good patient compliance and that patients need to finish the movement to expose the conjunctiva or their fingernail beds. Although this movement is quite easy for healthy people or patients with mild disease, it is difficult for severely anemic patients or those in a coma, compromising the research findings due to insufficient exposure to the characteristic areas. It can also be learned from the previous reports that after the collected data was filtered, it was necessary to eliminate some data whose feature areas were not fully exposed, leading to the reduction of the data (23). In this research, we collected the whole facial images for analysis instead of focusing on a specific organ or region of the face and used a deep learning model to automatically extract facial features to make a prediction. To explore the technical details related to facial feature recognition used by the proposed deep learning model, we used a technique called Gradient-weighted Class Activation Mapping (Grad-CAM) (35) to produce visual explanations for decisions of a convolutional neural network (CNN)-based model. Grad-CAM uses the gradients of any target concept, flowing into the final convolutional layer to produce a coarse localization map highlighting the important regions in the image for predicting the concept. The Grad-CAM results in Figures 8–10 demonstrate that the features extracted from eyes and lips contribute more to the prediction. Multiple facial features extracted from the videos assist the model in achieving better prediction performance compared with other models in previous reports. At the same time, the acquisition of facial images often only requires consent from patients or guardians without specific body positions or movement, which is convenient, reduces the potential harm to the patients, and is more in line with the requirements of medical ethics.

In this research, all the research equipment is a pad commonly used in public without the participation of additional auxiliary equipment. At present, a lot of auxiliary equipment is often used for image acquisition and analysis, such as a flash lamp, calibration card, etc. (36, 37). The use of these auxiliary equipment hinders the practical application of the experimental results. Each piece of additional auxiliary equipment adds an uncontrolled influencing factor to the research. Our research used only a pad to complete all video acquisition work. The videos were analyzed by artificial intelligence, realizing the rapid, convenient, and accurate detection of the anemic state, and the comparison result also showed promising clinical utility in the future. Promoting this deep learning-based anemia detection technology, especially in areas short of resources, will enable medical staff to quickly screen and detect the anemic state of patients with fewer resources.

Another strength of our research is that all the participants were patients in the critically ill area of the emergency department. Compared to previous research whose inclusion patients were clearly diagnosed and the diagnosis was simple or even just diagnosed as anemia, the diagnosis of inclusion patients in our research was more complicated, and the condition was more critical, such as gastrointestinal hemorrhage, severe trauma, acute myocardial infarction, and other emergencies. Some patients' diagnoses were combined with several diseases; some were even diagnosed with multiple organ dysfunction syndromes (MODS), and anemia was often diagnosed only after a routine blood test. Therefore, the facial changes of these patients were more complicated. In addition to the facial pallor and indifference caused by anemia, the facial changes caused by other diseases, such as the painful face caused by trauma and the face of comatose patients, would impact this research. The facial videos were analyzed by deep learning technology to screen for the features most relevant to anemia, contributing to the facial analysis model and establishing the prediction model of anemia from facial images with high accuracy.

In reality, detecting anemia via facial images is a part of our facial recognition research. In the emergency environment, patients' conditions are often complex and difficult to diagnose. The core point of triage is how to quickly judge the patient's condition and deploy the most appropriate treatment for them without wasting medical resources. Currently, the commonly used triage methods include “Simple Triage And Rapid Treatment” (START), “Abbreviated Injury Scale” (AIS), “Injury Severity Score” (ISS), and so on (38). Reasonably and accurately applying the triage methods often requires professional and experimental medical staff, which many medical institutions, especially in areas with limited resources, fail to meet the demand (39). Our research has realized the noninvasive, rapid, accurate detection of anemia for urgent cases of anemia. In the future, the application of our triage research will favor the quick judgment of patients' conditions to make reasonable triage decisions. Emergency and severely ill patients often exhibit different emergency faces, such as cyanosis and dyspnea of acute airway obstruction, breathing like dying patients, etc. We can make full use of these characteristics to establish a connection between the facial images and the critical degree of the patients so that we can find a new rapid and simple triage method. This will be a very complex but meaningful challenge for us.

Besides diagnosis and triage, our prediction model also has the potential to aid treatment. Many severely traumatic patients will appear in large-scale battlefield or mass casualty incidents where they may suffer from traumatic hemorrhagic shock, and timely blood transfusion treatment will significantly influence their prognosis (40). However, providing blood transfusion treatment to every patient with limited blood resources is impossible. Many patients suffer from severe trauma without obvious bleeding, such as closed abdominal or pelvic trauma. When there are not enough laboratory devices, it is difficult to diagnose the anemia state of these patients and whether they need an urgent blood transfusion. Our research achieved high accuracy in detecting severe anemia with the help of a portable device (Hb < 70 g/L), which was also the threshold of blood transfusion (41); it could aid doctors in the treatment decision for or against transfusion fast and accurately.

The limitation of this research is that we used a single pad to finish the research, and the changes in research results after using different devices or different deep learning technology may lead to the deviation of detection of anemia, which will be further verified in subsequent research. The validation set was from the same hospital, and multicenter validation is an important task in future studies. Our research participants were all Chinese, meaning the research results might not apply to white people or black people. Analyzing the imperfect results of our research, especially for mild and severe anemia, we attribute the limitations to two reasons: small facial changes in mild anemia and insufficient data. However, the promising results of this research convince us that further research with more data will bring us better results.

## Conclusion

Patients' anemia in the ED might be diagnosed fast and correctly by the machine learning prediction model, which would help physicians decide whether or not to administer a blood transfusion. It offers great clinical value and practical significance, expediting diagnoses, improving medical resource allocation, and providing appropriate treatment in the future.

## Data availability statement

The raw data supporting the conclusions of this article will be made available by the authors, without undue reservation.

## Ethics statement

Written informed consent was obtained from the individual(s) for the publication of any potentially identifiable images or data included in this article.

## Author contributions

AZ: research design and writing. JLo: analysis and model design. ZP: data review and supervision. JLu and HZ: data collection. XZ: data collection and validation. JLi: data review and methodology. LW: data review. XC: data validation. BJ: model design and supervision. LC: research supervision and article review. All authors contributed to the article and approved the submitted version.

## Funding

This research was supported by the National Key R&D Program of the Ministry of Science and Technology (2020YFB1313904).

## Conflict of interest

The authors declare that the research was conducted in the absence of any commercial or financial relationships that could be construed as a potential conflict of interest.

## Publisher's note

All claims expressed in this article are solely those of the authors and do not necessarily represent those of their affiliated organizations, or those of the publisher, the editors and the reviewers. Any product that may be evaluated in this article, or claim that may be made by its manufacturer, is not guaranteed or endorsed by the publisher.
